# 18F-Fluorodeoxyglucose-Positron Emission Tomography/Computed Tomography for Other Thyroid Cancers: Medullary, Anaplastic, Lymphoma and So Forth

**DOI:** 10.4274/mirt.60783

**Published:** 2017-02-01

**Authors:** Mine Araz, Derya Çayır

**Affiliations:** 1 Dışkapı Yıldırım Beyazıt Training and Research Hospital, Clinic of Nuclear Medicine, Ankara, Turkey

**Keywords:** thyroid cancer, Positron emission tomography, 18F-fluorodeoxyglucose

## Abstract

Positron emission tomography/computed tomography (PET/CT) with ^18^F-fluorodeoxyglucose (FDG) is used in staging, restaging, and evaluation of therapy response in many cancers as well as differentiated thyroid carcinomas especially in non-iodine avid variants. Its potential in less frequent thyroid tumors like medullary, anaplastic thyroid cancers, thyroid lymphoma and metastatic tumors of the thyroid however, is not well established yet. The aim of this review is to provide an overview on the recent applications and indications of ^18^F-FDG PET/CT in these tumors and to focus on the controversies in the clinical setting.

## Abstract (tr)

^18^F-fluorodeoksiglukoz (FDG) pozitron emisyon tomografisi/bilgisayarlı tomografi (PET/BT) pek çok kanserde ve özellikle iyot tutmayan varyant olmak üzere diferansiye tiroid karsinomlarında evreleme, yeniden evreleme ve tedaviye yanıtın değerlendirilmesinde kullanılır. Medüller, anaplastik tiroid kanserleri, tiroid lenfoması ve metastatik tümörler gibi tiroidin daha nadir tümörlerindeki potansiyeli henüz tam olarak belirlenmemiştir. Bu derlemenin amacı, ^18^F-FDG PET/BT’nin bu tümörlerdeki son uygulamaları ve endikasyonları hakkında genel bir bakış sunmak ve klinik çerçevedeki tartışmalar üzerine yoğunlaşmaktır.

## INTRODUCTION

^18^F-fluorodeoxyglucose (^18^F-FDG) is a metabolic positron emission tomography/computed tomography (PET/CT) imaging agent. Its uptake depends on the glycolytic rate of the tumor. Differentiated thyroid cancers have a relatively low metabolic rate with better differentiation and radioactive iodine avidity. Dedifferentiated and other types of thyroid cancers however, show a higher glycolytic rate and ^18^F-FDG uptake. ^18^F-FDG PET/CT now has a common role in staging and restaging of thyroid cancers other than differentiated subtypes. Although some other ^18^F and Ga-68 labeled radiopharmaceuticals (FDOPA and Ga-68 labeled peptides) have been introduced for the same indications, ^18^F-FDG PET/CT is still the PET radiopharmaceutical of choice with its wide availability and lower cost, and other PET tracers now only have a complementary role. This paper aims to focus on the role of ^18^F-FDG PET/CT in less common tumors of the thyroid.

## MEDULLARY THYROID CANCER

Medullary carcinoma is a neuroendocrine tumor of the thyroid originating from the parafollicular C cells. It is relatively infrequent and accounts for 5% of all thyroid cancers. It can present either as sporadic (75%) or familial (25%) forms. It characteristically secretes calcitonin (1).

Once medullary thyroid carcinoma is diagnosed by fine needle aspiration biopsy, neck ultrasound is recommended for preoperative lymph node assessment (2). For patients with preoperative calcitonin levels >500 pg/mL, additional radiologic imaging for evaluation of possible distant metastasis is indicated. Neck and chest CT, contrast-enhanced liver CT or magnetic resonance imaging (MRI), MRI of the axial skeleton and bone scintigraphy may be performed (3). ^18^F-FDG PET/CT is not routinely recommended at the staging of the disease (4).

Prognosis of the disease depends on the age and stage of the disease at diagnosis and the extent of the primary surgery performed. Additional factors for a better outcome in medullary thyroid cancer are female gender, well-differentiated histology, small tumor size, intracapsular tumor, lower levels of calcitonin in the postoperative period and absence of lymph node or distant metastasis. Although aggressive treatment strategies are followed in medullary thyroid cancer patients, persistent or recurrent disease is still frequently seen. Being the most sensitive tumor marker, serum calcitonin levels are usually elevated in recurrent cases. In approximately one third of the patients, carcinoembryonic antigen (CEA) is also increased. CEA elevation is also a prognostic determinant of advanced disease and dedifferentiation of the tumor (1,5). Biochemical recurrence leads the clinicians to investigate a tumor focus because tumor markers increase before radiologic examinations become positive. This is why identification of recurrent disease is quite problematic in disease management. In patients with postoperative calcitonin values <150 pg/mL, it is thought that recurrence is mostly related to locoregional disease and neck ultrasound is recommended. Serum calcitonin levels ≥150 pg/mL are more likely to be indicative of extensive disease (1). Functional imaging techniques have been found to be useful in this clinical setting (4,6,7,8). In a series of 55 medullary thyroid cancer patients with elevated calcitonin levels, the role of neck and abdomen USG, neck, chest, and abdomen CT, liver and whole-body MRI, bone scintigraphy, and ^18^F-FDG PET/CT were investigated comparatively. Conventional radiologic imaging methods were found to be more sensitive than ^18^F-FDG PET/CT for any site of tumor recurrence. They also found that maximum standardized uptake value (SUV_max_) levels were higher in patients with progressive disease, but there was a significant overlap with the stable cases. The authors concluded that ^18^F-FDG PET/CT had a low prognostic value in medullary thyroid cancer (9). However, opposing results have been obtained in some other studies (10,11,12,13,14).

There are several studies reported in this era investigating the role of ^18^F-FDG PET/CT in recurrent medullary thyroid cancer patients with high levels of tumor markers. The sensitivity and specificity of ^18^F-FDG PET/CT is reported in a wide range (10,11,12,13,14). In a large series of 100 examinations, Diehl et al. (15) have reported a sensitivity of 78% and specificity of 79%. A recent meta-analysis of the published data by Treglia et al. (7) reviewed 24 major studies. Despite the heterogeneity in the definition of true negative and false negatives, examination techniques and inclusion criteria in these studies, on a per patient pooled analysis, the authors calculated a detection rate of 59% [95% confidence interval (CI): 54-63%] for ^18^F-FDG PET or PET/CT in patients with suspected recurrent medullary thyroid cancer. The detection rate of ^18^F-FDG PET/CT was higher in advanced disease (for serum calcitonin levels <150 ng/dL; detection rate: 40%, 95% CI: 29-52%, ≥150 ng/dL; detection rate: 64%, 95% CI: 59-70%, and ≥1000 ng/dL; 75%, 95% CI: 67-81%) (7). Undetectability of recurrent tumors at low levels of calcitonin and CEA was mainly attributed to small tumor size or microscopic disease (16).

Detection rates were also found to be higher in patients with lower calcitonin and CEA doubling time (for calcitonin doubling time <12 months, detection rate: 76%, and for CEA doubling time <24 months, detection rate: 91%) (7). This is reasonable, as the tumors with higher rate of proliferation are expected to have a higher metabolic rate and increased glucose consumption and thus higher detectability with ^18^F-FDG PET/CT (8). However, aggressive forms of medullary thyroid cancer are not always seen in the clinical setting, more indolent cases are sometimes encountered. Such lesions express lower levels of ^18^F-FDG uptake with SUV_max_ values, previously reported as a mean (±standard deviation): 3.76±1.79. Skoura et al. (17) also reported an interesting finding that the sensitivity of ^18^F-FDG PET/CT for medullary cancer recurrence in patients with multiple endocrine neoplasia (MEN) type 2A was significantly lower (23%), and for calcitonin levels <2000 pg/mL sensitivity was calculated as 0% in MEN 2A patients. Excluding the patients with MEN 2A, the overall sensitivity of ^18^F-FDG PET/CT raised from 44.1% to 50%. Thus, the authors concluded that ^18^F-FDG PET/CT was more reliable in sporadic or MEN 2B patients (17).

Site of metastasis has also been recently reported to be important for detectability by ^18^F-FDG PET/CT. De Luca et al. (18) have retrospectively analyzed metastatic medullary thyroid cancer patients who had undergone ^18^F-FDG PET/CT. They concluded that ^18^F-FDG PET/CT was primarily useful in lymph node involvement evaluation. Lung, liver or brain metastases could be missed due to the small size or low metabolic activity and as for skeletal metastases, detectability was limited to lytic metastases (18).

Recently other PET radiopharmaceuticals have been suggested for use in detection of recurrence in medullary thyroid cancer patients. ^18^F-DOPA is reported to have a higher sensitivity as compared to ^18^F-FDG PET/CT, especially in more indolent disease (15,19,20,21,22,23,24). A recent study by Archier et al. (25) has also revealed that ^18^F-DOPA PET/CT was reliable for a compartment based approach in lymph node involvement. Gallium-68 labeled peptides are also under scope for this use, especially to identify patients with tumors positive for somatostatin receptors, and thus candidates for radionuclide therapy (26,27). ^18^F-FDG PET/CT should not be considered as the first step in the diagnostic algorithm and must be spared for the cases with elevated levels of tumor markers but negative conventional imaging examinations. One must also keep in mind that functional imaging methods are complementary to each other in the way that they all have different routes of radiopharmaceutical uptake and that they work efficiently under different circumstances (7,28,29,30).

## ANAPLASTIC THYROID CANCER

Anaplastic thyroid carcinoma is a rare malignancy constituting less than 2% of all thyroid cancers. It originates from follicular cells but is very poorly differentiated to have histopathologic characteristics of the differentiated tumors of the follicular cells. It shows a rapid growth and local invasion (31). Anaplastic thyroid carcinomas have a highly aggressive behavior with the worst prognosis among all thyroid cancers, given a median estimated survival of 6-8 months (1,32). This is why all anaplastic thyroid carcinomas are classified as Stage IV tumors and imaging methods for primary staging of the tumor includes CT of the head, neck, thorax, abdomen and pelvis as well as a bone scan or ^18^F-FDG PET/CT for identification of local or distant metastatic disease (1,33).

There is a limited number of studies evaluating ^18^F-FDG PET/CT in anaplastic thyroid carcinomas. However, ^18^F-FDG PET/CT may have a role in the follow-up of anaplastic thyroid cancer after primary surgery for detection of residual, recurrent, or metastatic disease (34,35). ^18^F-FDG PET/CT results are suggested to have an impact on the clinical management of anaplastic thyroid cancer. Anaplastic thyroid cancers have been reported to have a high glucose metabolism and thus, show a high ^18^F-FDG uptake. American Thyroid Association (ATA) have published a clinical management guideline on anaplastic thyroid cancer patients (4). ^18^F-FDG PET/CT has been recommended in anaplastic thyroid cancer at many steps in the management. It is recommended in the primary staging both for evaluation of lesion resectability and distant metastasis. Follow-up of anaplastic thyroid cancer patients is also successfully done by ^18^F-FDG PET/CT. It is recommended to be performed 3-6 months after therapy in patients either with or without persistent disease. ^18^F-FDG PET/CT is also recommended for distinction of anaplastic and differentiated thyroid cancer metastasis, based on the fact that anaplastic thyroid cancer metastases have a significantly higher SUV_max_ (4).

A few studies have investigated the possible prognostic role of ^18^F-FDG PET/CT in anaplastic thyroid cancer patients. As well as SUV_max_, metabolic tumor volume has been introduced as a new index. Because some tumors show non-homogenous uptake, metabolic tumor volume has been shown to be a more valuable parameter in various tumors (36). In a series reported by Bogsrud et al. (37), PET has affected management in about 50% of anaplastic thyroid cancer patients. It was also found that SUV_max_ and metabolic tumor volume had a prognostic significance in these patients (37). Poisson et al. (38) similarly concluded that a SUV_max_ >18 and a ^18^F-FDG uptake volume >300 mL had a significantly worse 6 month survival.

## THYROID LYMPHOMA

Thyroid lymphoma is a rare disease with female dominance, mostly seen in the elderly. It constitutes 1-5% of all thyroid malignancies and 1-2.5% of all lymphomas (39).

Primary thyroid lymphoma is most commonly B-cell originated, and 60-80% of all cases have been reported to be diffuse-large B-cell lymphomas. They are considered to arise from follicular cells (40,41). About 30% of the thyroid lymphomas is extranodal marginal zone lymphomas of mucosa-associated lymphoid tissue (MALT). They are usually associated with the existence of Hashimoto’s thyroiditis (42). Autoimmune stimulation is thought to be responsible for disease development.

There are only a few reports on the role of ^18^F-FDG PET/CT in primary thyroid lymphomas, and most of them are case presentations. This is partly due to the infrequency of the disease. In a case report presented by Naswa et al. (43), the authors showed that ^18^F-FDG PET/CT was useful in staging and detecting therapy response in high-grade primary thyroid lymphoma in a 64-year-old female patient with a history of Hashimoto’s thyroiditis.

On ^18^F-FDG PET/CT, Hashimoto’s thyroiditis is a primary problem in the differential diagnosis of lymphomas as both entities may show intense diffuse ^18^F-FDG uptake. MALT lymphomas have been reported to show false-negative results in ^18^F-FDG PET imaging (44). However, a recent study in a large patient group revealed that SUV_max_ was significantly higher and CT density was lower in primary thyroid lymphoma as compared to chronic thyroiditis. The authors have thus suggested that ^18^F-FDG PET/CT may be helpful in distinguishing primary thyroid lymphoma from chronic thyroiditis (45). Similarly, a large series of thyroid lymphoma from a single center have been recently reported. Among the defined radiologic characteristics in this retrospective analysis, high SUV_max_ levels were remarkable. They reported a median SUV_max_ of 22.7 (range: 10.6-37.6) (46). Riedel’s thyroiditis, an IgG4-related disease, also presents with diffuse thyroidal uptake and should be kept in mind in differential diagnosis (47).

## METASTATIC THYROID CANCER

Metastatic tumors of the thyroid are not uncommon (48,49). In autopsy series, the overall incidence of thyroid metastasis has been reported as 1.25% and as high as 24% in patients with known extensive disease metastatic to other sites (48,50). In a recent report of a large series, Hegerova et al. (49) listed the most common tumor types metastatic to the thyroid as follows: kidney (22%), lung (22%), and head and neck (12%). The role of ^18^F-FDG PET/CT in detection of metastatic tumors of thyroid is obscure. There are only a few case reports in the literature demonstrating the ^18^F-FDG uptake patterns of thyroid metastasis in other primary malignancies. Metastatic tumors of the thyroid are known to represent as thyroid masses, and thus they tend to show focal 18F-FDG uptake (51). However, Agrawal et al. (52) have recently defined heterogeneous thyroidal ^18^F-FDG uptake in a patient with known non-small cell lung carcinoma and multiple metastasis throughout the body. Any kind of unexpected thyroidal uptake in patients with known malignancies should be carefully evaluated for a possibility of thyroid metastasis.

## POORLY DIFFERENTIATED THYROID CARCINOMA

Poorly differentiated thyroid carcinoma histopathologically and behaviorally stands between differentiated thyroid carcinoma and anaplastic thyroid cancer. It does not appear de novo, differentiated tumors become de-differentiated by previously reported genetic alterations (53). Poorly differentiated thyroid carcinoma carries some characteristics of differentiation like thyroglobulin expression along with some features of anaplastic thyroid carcinoma like loss of iodine concentration ability and increased glucose transporter 1 (GLUT1) expression. The inverse relationship between iodine concentration and glucose uptake was defined as “flip-flop phenomenon” (54). Increased glucose uptake is partly related to the increased metabolic activity in highly proliferated tumor cells and Grabellus et al. (55) have also reported that de-differentiation was also accompanied by GLUT1 up-regulation. Increased glucose demand and GLUT expression provides high ^18^F-FDG uptake and detectability rates with ^18^F-FDG PET/CT. Compared to radioiodine scanning, poorly differentiated thyroid carcinoma are rather preferred to be screened by ^18^F-FDG PET/CT (56). It has a well documented role in all stages of disease evaluation. In preoperative staging, it has been reported to change management in 25% of the cases and that there was an inverse correlation between SUV_max_ levels and survival rates (57). In the postoperative period, Nascimento et al. (58) have recently recommended postoperative ^18^F-FDG PET/CT to be routinely performed in patients with aggressive histology. Usefulness of ^18^F-FDG PET/CT in the evaluation of therapy response was investigated. In anaplastic thyroid carcinoma patients who had undergone multimodal therapies, ^18^F-FDG PET/CT was suggested as a marker for treatment response (59).

## HURTHLE CELL CANCER

Hürthle cell cancer of the thyroid is a relatively uncommon form accounting for only 3.6% of all thyroid cancers (60). It has a worse prognosis and a higher tendency of metastases compared to other differentiated thyroid cancers (61,62,63). It especially has a more aggressive course when the primary tumor is widely invasive (64,65). It is known to have a lower radioiodine avidity (66,67). Thus, ^18^F-FDG PET/CT becomes a valuable alternative for imaging Hürthle cell cancer. The number of studies investigating the role of ^18^F-FDG PET/CT in Hürthle cell cancer is limited. A small report involving 12 patients found that ^18^F-FDG PET/CT was positive in 12 patients and that ^18^F-FDG PET/CT was the only positive imaging modality for localizing disease in 7/12 patients. Overall sensitivity was reported to be 92% and the patient management was changed by ^18^F-FDG PET/CT in 50% of all cases (68). In a heterogeneous group of thyroid cancer patients reported by Wang et al. (69), SUV_max_ and tumor volume were reported to have an important prognostic value in Hürthle cell cancer. Pryma et al. (70) reported 44 patients with Hürthle cell thyroid cancer. ^18^F-FDG PET/CT was performed for risk assessment after total thyroidectomy in patients with elevated thyroglobulin levels. They calculated the overall sensitivity and specificity of ^18^F-FDG PET/CT as 95% (70).

## THYROID INCIDENTALOMA

Thyroid incidentalomas are defined as thyroid lesions detected by an imaging study that were not previously suspected or detected in an asymptomatic patient. Incidental thyroidal ^18^F-FDG uptake is not uncommon in oncological ^18^F-FDG PET/CT studies performed for any other malignancy. As the use of ^18^F-FDG PET/CT in staging, restaging of other malignancies and evaluation of therapy response increased, the frequency of incidental ^18^F-FDG uptake has also increased. The incidence has been reported as 0.2-8.9% (71). There are many studies in the literature investigating the clinical significance of thyroidal ^18^F-FDG uptake. Overall malignancy rate in an incidentally detected thyroid lesion is in a wide range:13-59% (72) .The pattern of ^18^F-FDG uptake is important for evaluation of the etiology of the uptake. Diffuse thyroidal ^18^F-FDG uptake has been generally reported to be related to thyroiditis and autoimmune process, and it is seen in about 0.6-3.3% of all ^18^F-FDG PET/CT studies (73).

The frequency of focal incidentalomas are 0.2-10%. The risk of malignancy is known to be higher in the focal ^18^F-FDG uptake group as compared to diffuse thyroidal ^18^F-FDG uptake. However, the malignancy prevalence in patients who present with focal thyroidal ^18^F-FDG uptake differs in a wide range in the literature (8-64%) (74).

The most common type of thyroid cancer detected in thyroid incidentalomas on ^18^F-FDG PET/CT is papillary thyroid cancer and follicular type papillary thyroid cancer (81.1%). Primary thyroid lymphoma and thyroidal metastasis of other malignancies account for 4.1% of these (75). In another series reported by Agrawal et al. (76) however, the percentage of metastatic thyroid cancer or thyroid lymphoma incidentally detected were 44.4%. Some investigators have proposed that incidental thyroid malignancies tend to have a more aggressive histologic subtype and a higher tumor grade, and that they should be evaluated more carefully (77). This can be related with the increased ^18^F-FDG avidity in de-differentiated cancers, as described above.

First step in the evaluation of incidental thyroid nodules is thyroid ultrasound. But two studies revealed that ^18^F-FDG avid thyroid nodules are indicative of malignancy regardless of suspicious findings on ultrasound (78,79). Therefore, fine needle aspiration biopsy is strongly recommended in metabolically active thyroid nodules (80). The ATA has recently published a management guideline in thyroid nodules in adult patients. Thyroid incidentalomas detected by ^18^F-FDG PET/CT was also mentioned, and they recommended fine-needle aspiration biopsy for focal ^18^F-FDG uptake corresponding to a nodule on thyroid ultrasonography. Diffuse ^18^F-FDG uptake, accompanied by sonographic and clinical evidence of thyroiditis was not a necessity for histopathologic examination (81).

There have been many efforts to determine a SUV_max_ cut-off for thyroidal ^18^F-FDG uptake, above which will indicate malignancy. It is obvious that the SUV_max_ is statistically significantly high in malignant lesions but there is no safe cut-off value to guide management. In a study by Stangierski et al. (82), a total of 82 patients with focal ^18^F-FDG uptake in the thyroid were further investigated by fine needle aspiration biopsy. The mean SUV_max_ for benign lesions was calculated as 3.2 and for malignant lesions as 7.1. Because the range of SUV_max_ was between 1.4-17.5 in benign lesions and between 1.8-33.6 in malignant lesions, there was a wide overlap between the two subsets, and therefore no cut-off was reliable. Investigators also found out that for malignant lesions detected by ^18^F-FDG PET/CT, there was a significant correlation between the diameter of the nodule and SUV_max_. The authors have thus suggested a cautious examination in small lesions even if they have low ^18^F-FDG uptake.

SUV_max_ is not the only parameter to evaluate preoperative risk in incidental thyroid malignancies. Kim et al. (83) have investigated the role of total lesion glycolysis and metabolic tumor volume in the prediction of lateral lymph node metastasis in patients with incidentally detected differentiated thyroid carcinoma. They found out that these volume-based PET functional parameters were significant in predicting lateral lymph node metastasis. Risk stratification with these parameters may be of clinical value if supported by larger studies (83).

Functional imaging provides certain advantages which cannot be obtained by conventional anatomical imaging methods. In nuclear medicine practice, ^18^F-FDG now has a major place as it is a nonspecific marker of increased metabolism. Differentiated thyroid tumors usually have a silent course, while other tumors of the thyroid show a variable presentation. Dedifferentiation, poor differentiation and anaplastic characters of these tumors lean high ^18^F-FDG avidity. Considering all the aforementioned data, we recommend ^18^F-FDG PET/CT in both initial evaluation and follow-up of undifferentiated thyroid cancers. Medullary thyroid carcinoma, the neuroendocrine tumor of the thyroid, has other functional imaging options than ^18^F-FDG PET/CT like somatostatin receptor imaging. But, in advanced cases, especially with high levels of tumor markers, the tumor is most likely to be dedifferentiated and metabolic imaging, to the best of our experience, is the most accurate way to evaluate these patients. Thyroid lymphoma is a rare clinical condition and we have a limited clinical expertise, but recent publications revealed that this tumor presents with high metabolic rate and ^18^F-FDG PET/CT has a definitive role for thyroid lymphoma, primarily in cases with unexpected findings like rapidly growing neck mass, compression symptoms and weight loss.

## CONCLUSION

In medullary thyroid carcinoma; ^18^F-FDG PET/CT is not routinely recommended in the primary staging of the disease, but it has been reported to be useful in the follow-up to evaluate high levels of calcitonin and CEA. Detection rates have been found to be higher in shorter tumor marker doubling times and in sporadic cases as compared to MEN syndromes. Its prognostic significance is still under debate in medullary thyroid cancer. Limited data published on anaplastic thyroid carcinomas revealed that ^18^F-FDG PET/CT may have a role in both staging and follow-up of these patients. SUV_max_ and metabolic tumor volume values seem to have a prognostic importance. ^18^F-FDG PET/CT can be of value in the differential diagnosis of primary thyroid lymphoma and thyroiditis. Metastatic tumors of the thyroid are not as uncommon as previously assumed, so special attention should be paid on thyroidal ^18^F-FDG uptake in patients with known malignancies. In poorly differentiated thyroid cancers, it is reasonable to use ^18^F-FDG PET/CT for follow-up due to high ^18^F-FDG uptake and metabolic tumor rate. Hürthle cell cancer is a rather rare histopathologic subtype of thyroid cancer with less iodine avidity. ^18^F-FDG PET/CT seems to have an important role with high detection rates and sensitivity-specificity in Hürthle cell cancer. Incidental thyroidal ^18^F-FDG uptake necessitates further clarification, especially if focal uptake corresponds to a sonographically evident thyroid nodule.
